# Fast and accurate microRNA search using CNN

**DOI:** 10.1186/s12859-019-3279-2

**Published:** 2019-12-27

**Authors:** Xubo Tang, Yanni Sun

**Affiliations:** Department of Electronic Engineering, City University of Hong Kong, Kowloon Tong, Hong Kong SAR

**Keywords:** Convolution neural network (CNN), Deep learning, microRNA, Open set problem

## Abstract

**Background:**

There are many different types of microRNAs (miRNAs) and elucidating their functions is still under intensive research. A fundamental step in functional annotation of a new miRNA is to classify it into characterized miRNA families, such as those in Rfam and miRBase. With the accumulation of annotated miRNAs, it becomes possible to use deep learning-based models to classify different types of miRNAs. In this work, we investigate several key issues associated with successful application of deep learning models for miRNA classification. First, as secondary structure conservation is a prominent feature for noncoding RNAs including miRNAs, we examine whether secondary structure-based encoding improves classification accuracy. Second, as there are many more non-miRNA sequences than miRNAs, instead of assigning a negative class for all non-miRNA sequences, we test whether using softmax output can distinguish in-distribution and out-of-distribution samples. Finally, we investigate whether deep learning models can correctly classify sequences from small miRNA families.

**Results:**

We present our trained convolutional neural network (CNN) models for classifying miRNAs using different types of feature learning and encoding methods. In the first method, we explicitly encode the predicted secondary structure in a matrix. In the second method, we use only the primary sequence information and one-hot encoding matrix. In addition, in order to reject sequences that should not be classified into targeted miRNA families, we use a threshold derived from softmax layer to exclude out-of-distribution sequences, which is an important feature to make this model useful for real transcriptomic data. The comparison with the state-of-the-art ncRNA classification tools such as Infernal shows that our method can achieve comparable sensitivity and accuracy while being significantly faster.

**Conclusion:**

Automatic feature learning in CNN can lead to better classification accuracy and sensitivity for miRNA classification and annotation. The trained models and also associated codes are freely available at https://github.com/HubertTang/DeepMir.

## Introduction

Non-coding RNAs (ncRNAs) refer to the RNAs that do not encode proteins and function directly as RNAs. Genome annotation of many different genomes show that ncRNAs are ubiquitous and have various important functions [1]. Besides commonly seen house-keeping ncRNAs such as transfer RNAs (tRNAs), ribosome RNAs (rRNAs), many small ncRNAs play important roles in gene regulation. This work is mainly concerned with a type of small ncRNA, microRNA (miRNA), which act as key regulators of gene expression at post-transcriptional level in different species [2–5]. In metazoans, mature miRNAs bind to the 3’-UTR of target mRNAs and can repress translation or promote mRNA degradation. As an miRNA can bind to multiple mRNA transcripts, a large number of protein-coding genes can be regulated by miRNAs [6, 7].

Because miRNAs’ important functions and their associations with complicated diseases in human, there are intensive research about miRNA gene annotation, target search, function identification etc. A fundamental step in miRNA research is the identification of miRNA genes in genomes. In the canonical miRNA biogenesis pathway, miRNAs are processed from longer transcripts named as primary miRNAs (pri-miRNAs) [3]. The hairpin structures of pri-miRNAs are cleaved by a member of RNase II family of enzymes, Drosha and produce precursor miRNA (pre-miRNA) in the nucleus [8, 9]. Pre-miRNAs are then exported to the cytoplasm, where Dicer cleaves off the loop region of the hairpin and further processes it to mature miRNA(s) of about 21 nucleotides [10, 11]. MiRNA gene annotation usually refers to identification of pre-miRNAs and mature miRNAs.

Existing miRNA annotation tools can be generally divided into two groups depending on whether reference miRNA genes are used. Homology-based miRNA search identifies pre-miRNAs by conducting sequence and/or secondary structural similarity search against existing miRNA genes. Like other ncRNAs, pre-miRNAs preserve strong secondary structures [2]. Thus, homology search models [12, 13] that can explicitly encode both sequence and structural similarities usually achieve high sensitivity and accuracy in classifying query sequences into their originating homologous families. However, the high sensitivity comes with a price of high computational cost. For example, structural homology search models based on context-free grammar have cubic running time complexity [14]. Even with various heuristic filtration techniques, it can be still very time-consuming to conduct large-scale sequence classification using both sequence and structural alignments. Sequence similarity-based homology search tools such as BLAST [15] can be also applied to classify pre-miRNAs to their native families. However, remote homologs with high structural but low sequence conservation tend to be missed. Another group of tools [16–18] do not use reference sequences for pre-miRNA search. These de novo miRNA search methods mainly use features such as hairpin structures of pre-miRNAs to identify putative pre-miRNAs in genomes. As a large number of regions in a genome can form hair-pin structures, features from RNA-Seq [19] data such as expression levels and read mapping patterns are often used to reduce the false positive rate of miRNA search [20–23]. Both types of tools are useful for miRNA search and annotation. De novo methods have the advantage of identifying possibly novel miRNAs but additional processing is needed to validate the findings.

Homology search-based miRNA search methods can take advantage of accumulating characterized miRNAs. For example, MiRBase [24] is an online database for miRNA sequences and annotation. The current release 22 contains 1983 miRNA families from 271 organisms, including 38,589 pre-miRNAs and 48,860 mature miRNAs. Rfam [25] is a comprehensive ncRNA family database with over 3,000 ncRNA families. The release 14.1 contains 529 pre-miRNA families and 215,122 precursor sequences.

These classified pre-miRNA sequences can be used as training data for deep learning based models. Depending on the choice of the training sequences and the design of the model architecture, deep learning-based miRNA search can be applied to distinguish miRNAs from other types of ncRNAs and also to conduct finer scale classification for different types of miRNAs. In this work, we explore whether using convolutional neural network (CNN) has advantages in distinguishing different types of miRNAs over powerful covariance models. In particular, we investigated how the input sequence encoding and training set construction affect the performance of miRNA characterization using CNN.

We choose CNN as the deep learning model because of its recent success in other sequence classification studies [26–29]. Empirical analyses have shown that CNN can be applied to extract “motifs” from a set of homologous sequences. Motifs are essential features to distinguishing different groups of sequence families including miRNAs. DeepBind [26] used a single convolution layer to capture the motif from protein binding sites. DeepFam [29] applied the CNN on the protein classification and found that the frequently activated convolution filters are consistent with known motifs. As different miRNA families tend to have different conserved sequences, the convolution layers in CNN are expected to capture distinctive features for fine-grained classification. DanQ [30], proposed by Qiang et al., added additional long short term memory (LSTM) layers above the convolution layers to capture the dependency between the separated motifs extracted by convolution layers. But as miRNAs are relatively short, the sequential features within a filter are sufficient for classification.

### Related work

In this section, we summarize related work on homology search-based miRNA identification. Some homology search tools are designed for comprehensive ncRNA search and can divide miRNAs into different types. For example, there are hundreds of different miRNA families in Rfam. The associated tool, Infernal [12], conducts homology search by incorporating both sequence and secondary structure similarities in context-free grammar based models. Input sequences can be classified into different miRNA families for functional inference. For identifying miRNAs with high sequence similarity, generic homology search tools such as BLASTn [15] can be applied as well.

Most tools designed specifically for miRNA search aim to distinguish miRNAs from other types of sequences [31–33]. The most successful ones usually employ transcriptomic data to improve the identification accuracy. When the reference genomes are available, reads from small RNA-Seq data are mapped to the reference genomes to locate possible pre-miRNA genes. Features such as the conserved hairpin structure, read mapping patterns on the mature miRNA vs. other regions, expression levels across multiple samples are utilized to screen miRNAs in those candidate regions. From the perspective of machine learning, distinguishing miRNAs from other regions can be formulated as a binary classification problem. Pre-miRNAs have the positive label and all others have the negative label. Classification models such as SVM [34, 35], Random Forest [36], and CNN [37] have been applied for miRNA search. Being different from these binary classification tools, ours focuses on classifying input sequences into different miRNA families for more detailed function annotation. Unrelated sequences including other types of ncRNAs are rejected using a threshold in the softmax value.

CNN was also employed by Genta Aoki [38] for ncRNA classification. The authors took ncRNA pairwise alignments and associated features as input to CNN and got 98% accuracy for 6 types of ncRNA.

Advances of feature selection and classification models in machine learning have enhanced the sensitivity and precision for miRNA search. However, highly unbalanced training set is still a challenge for various learning models [39]. Being formulated as a binary classification problem, there are significantly more negative samples (non-miRNAs) than positive samples (miRNAs). In addition, there are many different types of non-miRNA sequences. It is not clear how to compose the negative training data from such large and highly diverse sequences.

In this study, we intend to formulate miRNA search as a multi-label classification problem. Instead of using non-miRNAs as training data, we reject those un-relevant sequences using methods from open set problem [40]. In addition, we implemented two types of encoding methods based on whether we explicitly encode the secondary structure information.

## Method

The deep learning model we choose is Convolutional Neural Network (CNN), which has demonstrated some success in ncRNAs classification [38]. We implemented and compared two different encoding methods for CNN-based miRNA classification. In the first encoding method, we explicitly encode secondary structure information into matrices and use these matrices as training/testing data. In the second method, we use one-hot encoding matrix to represent the input sequences and do not take into account predicted secondary structures.

### Explicitly encode secondary structures into matrices

We implemented three types of matrix to encode the secondary structure information from sequences: **probability matrix, pair matrix, and mixed matrix**. The first two are inspired from adjacency matrix for modeling secondary structures. The structural information is derived from the sequences using RNAfold [41], which is one module in the ViennaRNA [41] package. As the optimal structure predicted based on Minimum Free Energy (MFE) is often not accurate, we use RNAfold to output both the optimal and suboptimal structures. In addition, we also use the base pairing probabilities computed by the software.

***Probability matrix*** simply contains the values of the base pairing probability outputted by RNAfold. For a sequence *s*, the size of the matrix is |*s*|×|*s*|. *P*_*i*,*j*_ is the predicted base pairing probability between the *i*th and *j*th base in *s* if the probability *p* is above a given threshold *T*. The equation for defining the value of each cell can be found below.

$$P_{i,j(probability\ matrix)} =\left\{ \begin{array}{rcl} p & & {if\ p\ \geq\ T}\\ 0 & & {if\ p\ <\ T.} \end{array} \right. $$

Being different from probability matrix, ***pair matrix*** distinguishes different base pairs including Watson-Crick pairs and G-U pair. If the base pairing probability is above a given threshold, we will record this base pair using its ID number, which is used to distinguish different base pairs. Depending on whether we take into account the order of the bases in a base pair, different base pairs can be converted into 6 or 3 different values. The conversion rules are summarized in the following equations. *X*_*i*,*j*_ refers to an element at position (*i*,*j*) in a pair matrix. *s*_*i*_ refers to the *i*th base in sequence *s*. *T* is a given threshold.

$$ X_{i,j(pair\ matrix\ with\ order)} = \left\{\begin{array}{ll} 0, &\text{if } {p} < \mathrm{T} \\ 1/6, &\text{if}\ (s_{i} s_{j}=AU)\ \text{and}\ p \geq \mathrm{T} \\ 2/6, &\text{if}\ (s_{i} s_{j}=UA)\ \text{and}\ p \geq \mathrm{T} \\ 3/6, &\text{if}\ (s_{i} s_{j}=CG)\ \text{and}\ p \geq \mathrm{T} \\ 4/6, &\text{if}\ (s_{i} s_{j}=GC)\ \text{and}\ p \geq \mathrm{T} \\ 5/6, &\text{if}\ (s_{i} s_{j}=GU)\ \text{and}\ p \geq \mathrm{T} \\ 6/6, &\text{if}\ (s_{i} s_{j}=UG)\ \text{and}\ p \geq \mathrm{T} \end{array}\right. $$

or

$${\begin{aligned} X_{i,j(pair\ matrix\ without\ order)}\! =&\\ &\left\{\begin{array}{ll} \!0, &\text{if } {p} < \mathrm{T} \\ \!1/3, &\text{if}\ (s_{i} s_{j}\,=\,AU \text{or}\ s_{i} s_{j}\,=\,UA)\!\ \text{and}\ p \geq \mathrm{T} \\ \!2/3, &\text{if}\ (s_{i} s_{j}\,=\,CG \text{or}\ s_{i} s_{j}\,=\,GC)\!\ \text{and}\ p \geq \mathrm{T} \\ \!3/3, &\text{if}\ (s_{i} s_{j}\,=\,GU \text{or}\ s_{i} s_{j}\,=\,UG)\!\ \text{and}\ p \geq \mathrm{T} \end{array}\right. \end{aligned}} $$

**Fig. 1:**
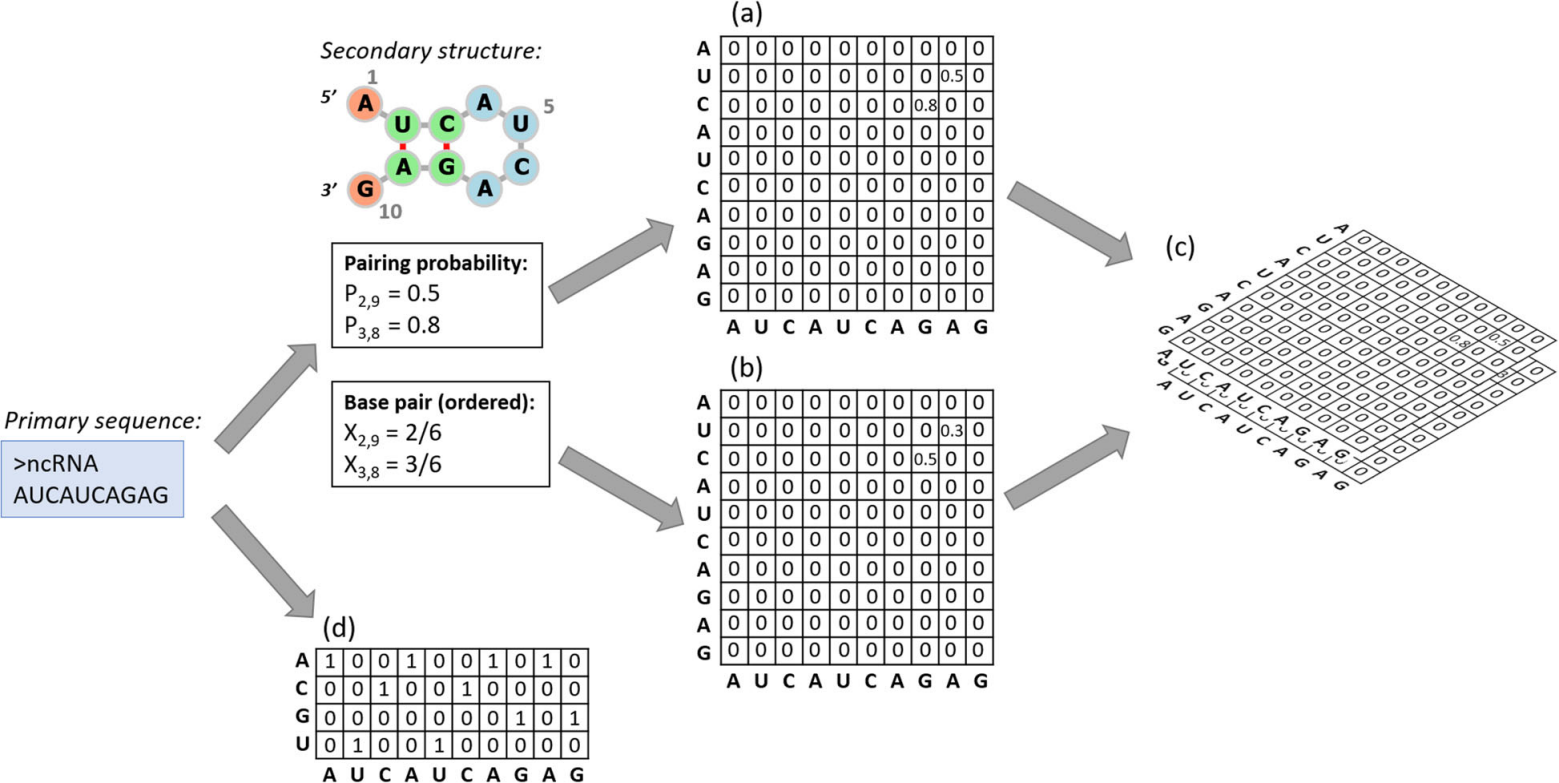
Examples of different encoding matrices. (**a**) Probability matrix; (**b**) Pair matrix; (**c**) Mixed matrix; (**d**) One-hot encoding matrix

Combining these two features together, the original 2D matrix will become a 3D matrix with two layers, which is called ***mixed matrix***, as shown in Fig. 1c. One layer of size |*s*|×|*s*| is the probability matrix and another layer of the same size is the pair matrix. Essentially, this matrix integrates different base pairs with the predicted pairing intensities.

**Fig. 2:**
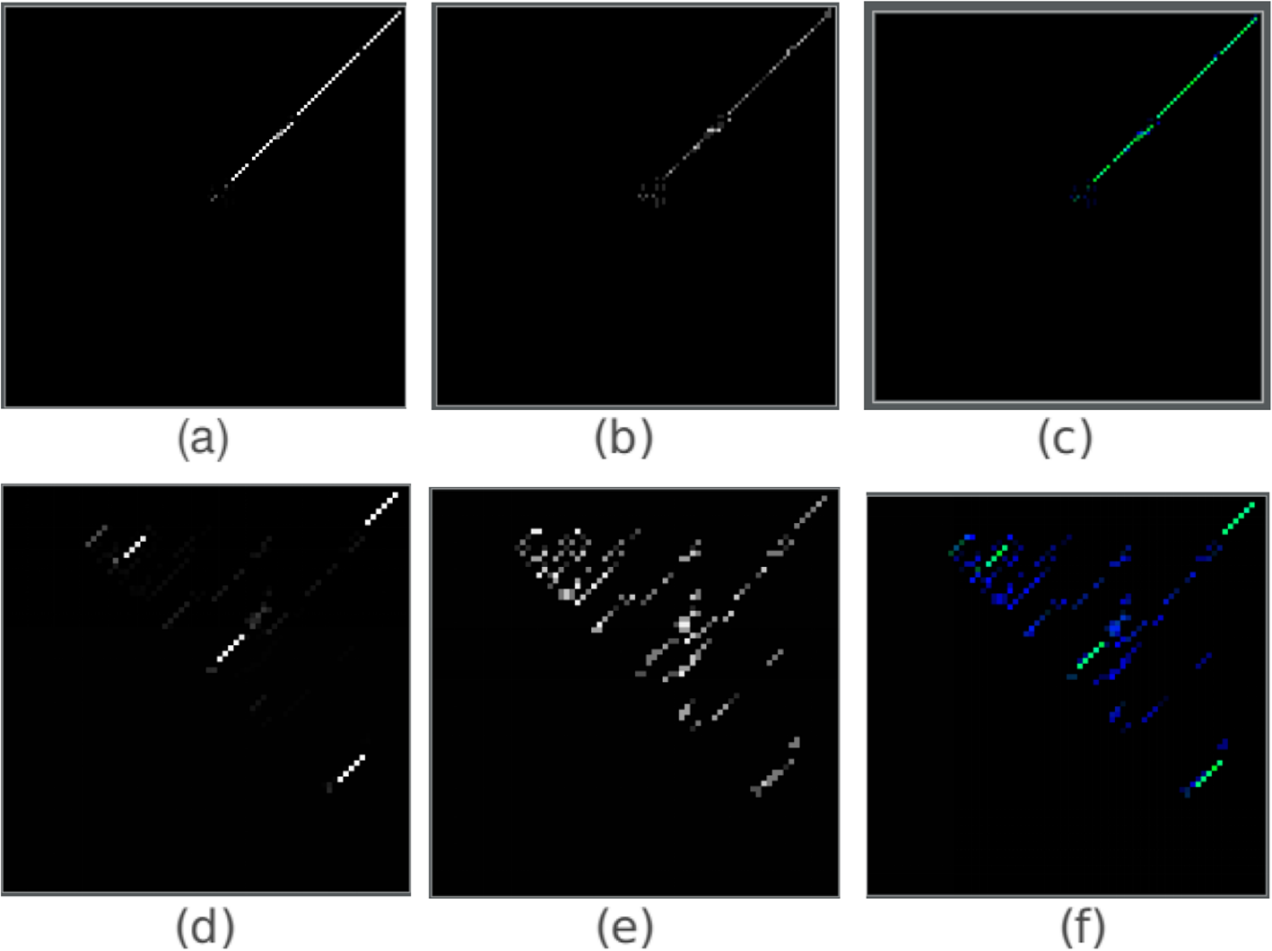
The probability, pair and mixed matrix images of miRNA and tRNA. (**a**), (**b**), (**c**) correspond to probability matrix, ordered pair matrix, mixed matrix of a miRNA sequence respectively. (**d**), (**e**), (**f**) correspond to probability matrix, ordered pair matrix, mixed matrix of a tRNA sequence respectively. For the mixed matrices, the color green is from the layer of probability matrix while blue represents the layer of the pair matrix

The pair and mixed matrices can be conveniently visualized as images. We presented the corresponding images for one miRNA and one tRNA in Fig. 2. The threshold *T* is 0.0001 in all the matrices. It is not hard to observe the stacking base pairs of the hairpin and cloverleaf structures of the miRNA and tRNA, respectively. The secondary structures are less obvious in the pair matrix because the cell values in the pair matrix are decided by the base pairs rather than the base pairing probabilities. Given a small *T*, cells with low pairing probabilities might still get a relatively big value because of the conversion rules.

#### CNN architecture for the matrices containing base pairing information

**Fig. 3:**
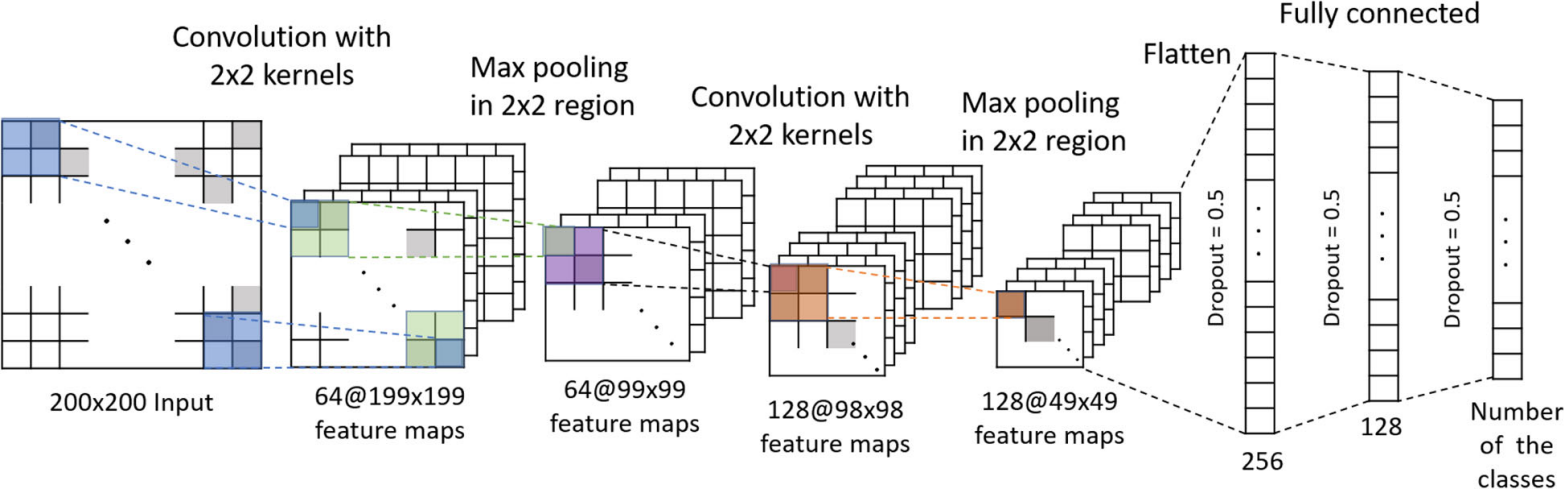
CNN structure of the probability/pair/mixed matrix

The CNN model contains two convolutional layers, followed by max pooling layers and three fully connected layers. Figure 3 sketches this architecture. To prevent overfitting, dropout is also applied. During the training of the CNN model, several hyperparameters were tuned within the given ranges, which are shown in Table 1. The parameters with best performance were selected. Finally, the hyperparameters were set as follows: number of convolution layers = 2, kernel size for each convolution layer = 2, the number of kernels in the two convolution layer = 64: 128, pooling method = max pooling, number of units in two fully connected layer = 256: 128, learning algorithm = Adam, dropout rate = 0.5, learning rate = 0.001, batch size = 32. The CNN model was implemented in Keras [42].

**Table 1:** The list of the tuned hyperparameters

Hyperparameter	Prob/pair/mixed matrix	One-hot matrix
Number of convolution layers	2, 4, 6	1
Kernel size for convolution	2, 3, 5	[8, 16], [4, 8, 12, 16], [2, 4, 6, 8, 10, 12, 14, 16]
		[2, 4, 6, 8, 10, 12, 14, 16]
		[2, 4, 6, 8, 10, 12, 14, 16]
Number of kernels (1st convolution layer)	16, 32, 64	64, 128, 256, 512
Number of kernels (2nd convolution layer)	32, 64, 128	not applicable
Pooling method	Max pooling, average pooling	
Number of units (1st fully connected layer)	64, 128,256	128, 256, 512
Number of units (2nd fully connected layer)	32, 64, 128	not applicable
Learning algorithm	Adam, SGD	
Dropout rate	0.7, 0.5	

### Encoding the sequence using one-hot matrix

One-hot encoding matrix has been successfully used in encoding genomic sequences for deep learning models. Essentially, the sequence is converted to a |*s*|×4 one-hot encodidng matrix, where |*s*| is the length of an input sequence and 4 is the number of different bases. Let the matrix be *M*, where *M*_*i*,*j*_ is 1 if the *i*th base in the input sequence is the *j*th character in the alphabet. For any other characters, *M*_*i*,*j*_ is 0 (*k*≠*j*). An example one-hot encoding matrix is given in Fig. 1d.

#### The CNN architecture for one-hot encoding matrices

**Fig. 4:**
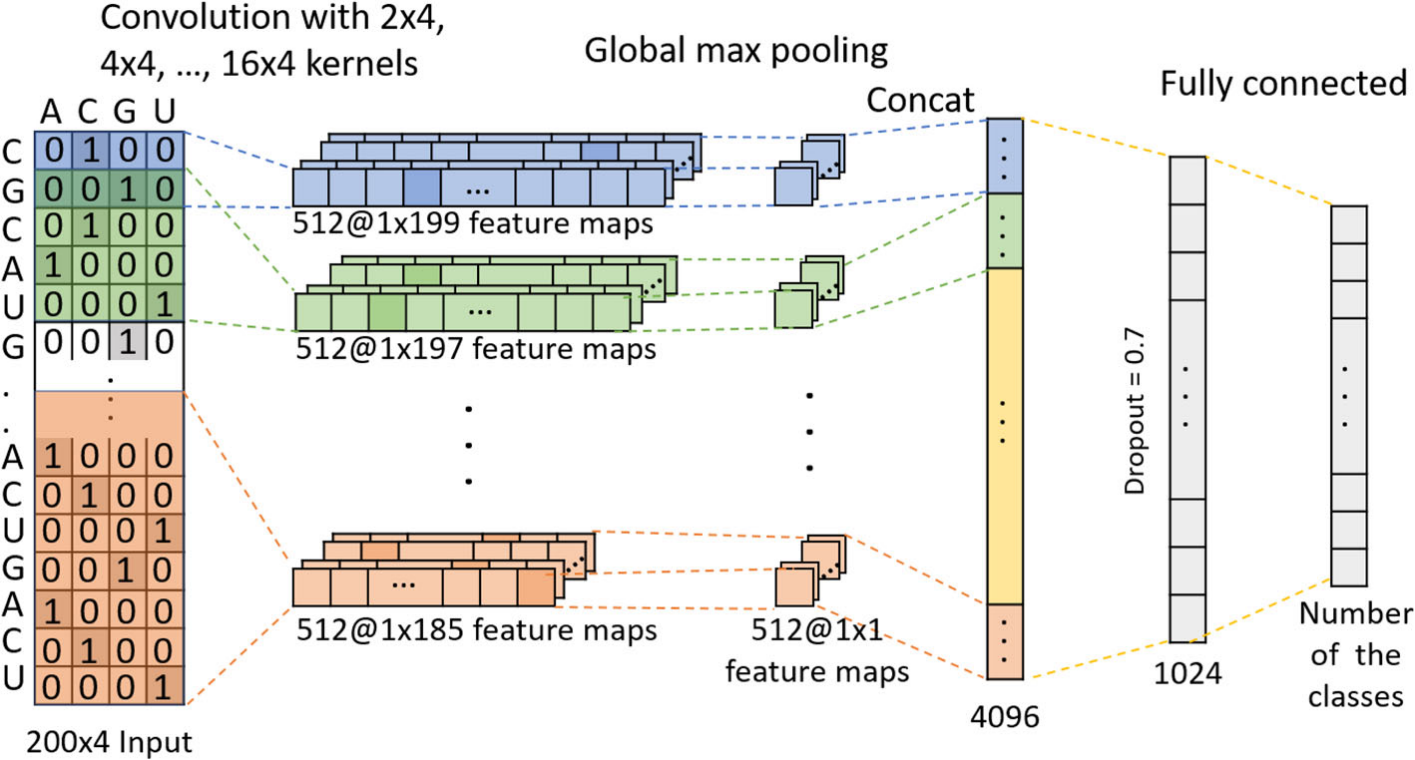
The CNN architecture of the one-hot encoding matrix encoding method

Inspired by Yoon Kim’s work in sentence classification [43], a similar model is used in this work. Several convolution layers with different size of kernels, followed by global max pooling layer, are connected to input layer directly. The outputs of all pooling layers are concatenated together and then fed into two fully connected layers. Dropout is also employed to overcome overfitting. Tuned parameters are shown in Table 1. Finally, the hyperparameters are set as follow: the number of convolution layers = 1, the size of the convolution filters = [2, 4, 6, 8, 10, 12, 14, 16], the number of kernel in convolutional layer = 512, the number of units in first fully connected layer = 1024, dropout rate = 0.7, learning rate = 0.001, learning algorithm = Adam, batch size = 64. Figure 4 shows the architecture.

### Excluding other ncRNA sequences using softmax probability threshold

As next-generation sequencing data such as small RNA-Seq data have become the major source of new miRNA discovery, useful miRNA search tools should be able to distinguish miRNAs from other types of ncRNAs, which usually co-exist with miRNAs in RNA-Seq data. Identifying miRNAs in RNA-Seq data is open set and thus any useful system must reject unknown/unseen classes in test set [40]. Existing binary classification tools often treat all the non-miRNA sequences as negative and need to choose non-miRNAs as the negative training samples. This often creates a highly unbalanced training set because there are significantly more non-miRNAs than miRNAs. In addition, it is not clear how to sample negative training sequences from many different types of ncRNAs. Our CNN model does not use an extra label for other ncRNAs. Instead, we reject out-of-distribution samples using the probability output of the softmax layer [44].

There are previous studies showing that the softmax probabilities of out-of-distribution samples are smaller than the probabilities of targeted samples [44]. Intuitively, out-of-distribution queries tend to produce a softmax probability vector with similar (small) values while an in-distribution query often yields a large softmax probability for one class. Thus, we will use carefully chosen softmax probability threshold to reject out-of-distribution samples, which in our case can be other types of ncRNAs in small RNA-Seq data. In addition, not all miRNA families are used in our training data. Any unseen miRNA families are also out-of-distribution samples. The softmax probability threshold should be used to reject them as well. We will use ROC curves to empirically choose a threshold.

## Experimental results

We will first compare the classification accuracy of the two types of encoding methods. In particular, we will examine whether explicitly encoding the structural information in input matrices can improve the performance of miRNA classification. As real data such as small RNA-Seq data contain different types of transcripts, we will examine whether the softmax output can be used to reject non-miRNA sequences. Then, we will compare the performance of the CNN-based miRNA classification with other ncRNA classification tools.

### Experimental data and pre-processing

For most of our training process, we use pre-miRNA families from Rfam as the training and testing data because we would like to compare our method with Infernal [12], which can conveniently use trained covariance models from Rfam. The current release of Rfam contains 529 pre-miRNA families and 215,122 precursor sequences. Another popular miRNA database is miRBase [24], which currently contains 1983 miRNA families from 271 organisms, including 38,589 pre-miRNAs and 48,860 mature miRNAs. In the experiment where we only use the mature miRNAs as the training data, we use miRBase because miRBase provides easy access to collect all the mature miRNAs.

**Fig. 5:**
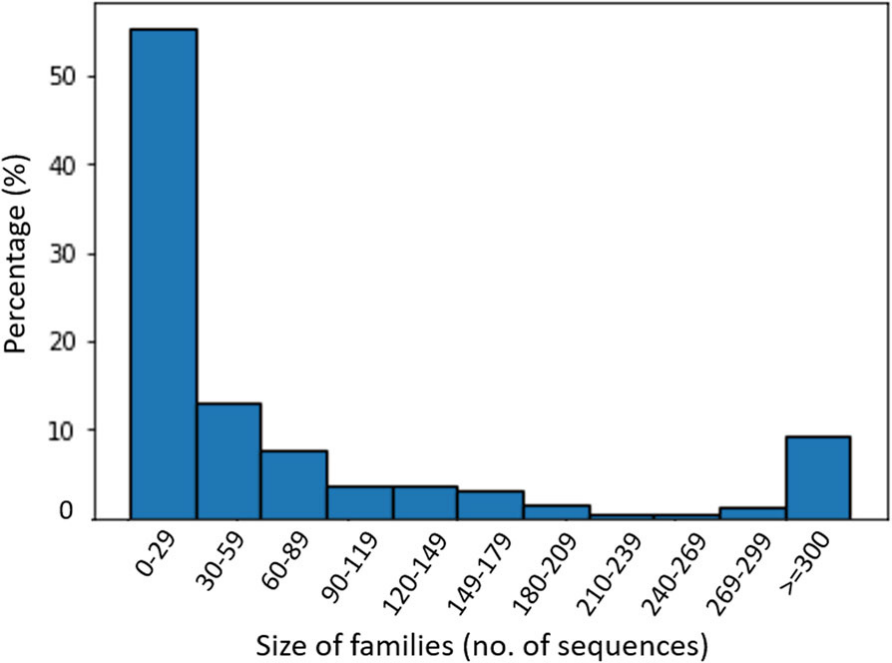
Rfam characteristics. Percentage of families in family size

We noticed that some of the pre-miRNA families in Rfam contain repeated sequences. Thus, in our pre-processing step, we will remove all the redundant sequences from the 529 pre-miRNA families in Rfam. As a result, 17.6% sequences were removed and 177,160 sequences were kept for downstream analysis. Each family contained different number of sequences (from 1 to 95,247) with different length. The distribution of the family size is shown in Fig. 5.

To train in mini-batch, a fixed size of the input matrix should be set. Although there are a few pre-miRNA families with particularly long sequences, 96.88% miRNAs in Rfam were less than 200nt. Thus, we only keep the families with size at most 200nt. Although commonly seen pre-miRNAs are about 70nt, we did not exclude the long ones, such as those occurring in plant genomes, before pre-processing. The input matrix has size 200. All the shorter sequences were converted into 200nt sequences by inserting zero padding at the end. These padded zeros will lead to zero during the scanning of a convolution filter and thus won’t affect the downstream layers after maxpooling.

### Classification performance of probability and pair matrix

Following our definition of the probability and pair matrix, a threshold *T* is needed to decide the values of these matrices. In this experiment, we evaluate the change of *T* on the classification performance. At the same time, we also compare the performance of ordered and unordered pair matrices. These experiments were conducted using 30 randomly selected pre-miRNA families with at least 100 member sequences.

Considering that the probabilities may not be linearly distributed from 0 to 1, we sorted all the pairing probabilities (greater than 0.0001) of each miRNA sequence in Rfam and then used the values of different percentiles as the thresholds. The 0th, 10th, 20th, 30th and 40th percentile are selected; the corresponding values are 0.0001, 0.00487, 0.00772, 0.01307, and 0.02411.

**Fig. 6:**
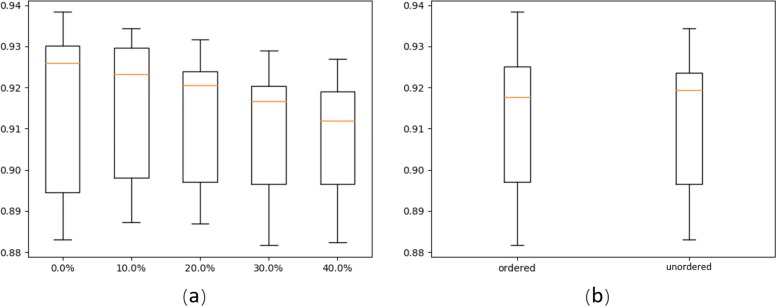
Performance comparison on classification accuracy using different secondary structure encoding methods. **a** 5 different thresholds (*T*) of base pairing probabilities. **b** ordered vs. unordered base pairs

For the 30 pre-miRNA families, 100 sequences were randomly selected from all member sequences. Then we used 5-fold cross validation so that there were 80 training sequences vs. 20 test sequences. CNN models with 30 classes are trained using different types of encoding methods. As there are 10 different types of matrices using 5 thresholds combined with two types of base pairs (ordered vs. unordered), 10 CNNs are trained. Note that the test sequences are encoded using the same method as the corresponding training data. We first compared the classification accuracy of using different thresholds with boxplot in Fig. 6a. For each threshold, there are 10 classification accuracy values for 5-fold cross validation results of both ordered and unordered cases. The comparison shows that allowing small base pairing probabilities yields higher average accuracy but also a slightly larger deviation. Overall, because of the higher average accuracy, we set the default threshold *T* as 0.0001 in all the following experiments. Figure 6b compares the classification accuracy of ordered vs. unordered matrices. The results show that they have very similar accuracy, with median accuracy around 0.92. By default, we use ordered base pairs in the pair matrix.

### Performance on pre-miRNAs classification

One-hot encoding matrix has been widely adopted for converting genomic data as inputs to deep learning models. Although it does not explicitly incorporate any structure information from the sequences, it has successful applications in protein homology search [29]. Thus, we will conduct a comprehensive experiment to compare the performance of one-hot encoding matrix and probability/pair matrix using pre-miRNA families from Rfam.

As different pre-miRNA families have different numbers of sequences, which can affect the performance of classification, we built 4 different datasets based on the size of families. Each dataset has different number of “classes” or “labels”. The details about the four groups can be found in Table 2. Taken the Rfam-300 dataset as an example, there are 47 families in this dataset and each family contains 300 sequences (including 250 training sequences and 50 testing sequences). The model trained using this dataset needs to classify queries into one of the 47 families (or classes). We will compare the classification performance of CNNs on the four groups of training data and examine how the training set size affects the accuracy.

**Table 2:** Four groups of pre-miRNA families with different training set sizes

Datasets	No. of families (i.e. classes)	No. of sequences per family (*train*:*test*)
Rfam-300	47	300 (250:50)
Rfam-120	106	120 (100:20)
Rfam-60	165	60 (50:10)
Rfam-30	241	30 (25:5)

In order to quantify the prediction performance, we use two metrics: accuracy and F-score $\left (F-score = \frac {2 \times Precision \times Recall}{Precision+Recall}\right)$. Classification accuracy quantifies the percentage of the correct predictions in all the test sequences. For each family, we also computed the recall $\left (Recall=\frac {TP}{TP+FN}\right)$ and precision $\left (Precision=\frac {TP}{TP+FP}\right)$. Here, TP, TN, FP, and FN correspond to the numbers of true positive, true negative, false positive, and false negative, respectively. The average F-score for all different families for one trained CNN is reported in Table 3. We evaluated the performance by the average accuracy of 5 independent experiments, each of which was measured with randomly selected testing sequences.

**Table 3:** Prediction accuracy(%) and F-score(%) of CNNs trained on families of different sizes

Method	Rfam-300		Rfam-120		Rfam-60		Rfam-30	
	Acc.^1^	F-score	Acc.	F-score	Acc.	F-score	Acc.	F-score
Pair matrix	89.91	89.99	77.71	76.87	71.27	68.82	60.60	56.32
Prob matrix	83.69	83.28	72.86	71.61	69.83	67.66	59.37	54.72
Mixed matrix	87.78	87.32	74.94	73.67	66.15	63.67	53.31	48.71
One-hot matrix	99.25	99.25	98.87	98.88	98.48	98.45	97.76	97.71

^1^Acc. refers to accuracy (%)

The results show that using one-hot encoding matrix led to much better performance than other methods even though it does not integrate base pairing information. In addition, it was less susceptible to the reduction of training data size. On the other hand, matrices focusing on base pairs need bigger training data to achieve better classification accuracy. These comparisons indicate that using one-hot encoding matrices is able to distinguish different types of miRNA families. One possible reason behind the inferior performance of using base pairing information is that all these pre-miRNA families have similar secondary structures and thus it is more difficult to conduct finer scale classification within the big family of miRNAs. For using one-hot matrix is less vulnerable to the decreased size of the training dataset, one possible reason is that one-hot matrix model has much fewer trainable parameters. For example, inputting the same sequence of length 200nt, one-hot model can update 4,485,255 parameters while the pair matrix model can update 78,748,399 parameters. Fewer parameters can help the model maintain high accuracy even if the training set is relatively small.

However, our additional experiments (next section) showed that these matrices cannot distinguish miRNAs from C/D box snoRNAs with high accuracy either, probably because of the similarity in the secondary structures, indicating that it is more difficult to train effective CNNs for matrices encoding base pairs. Larger training data are needed to improve the classification accuracy, which may not be always available for some miRNA families.

### Use softmax probability threshold to reject other types of ncRNA sequences

Transcriptomic data such as small RNA-seq data can contain reads from other types of ncRNAs or miRNA families that are different from the many data. In this experiment, we will show that appropriate softmax probability value can be chosen as the threshold to distinguish targeted miRNAs from out-of-distribution samples.

As an example, we demonstrate the softmax output using the CNN model trained on Rfam-60 dataset (including 165 miRNA families). The positive set includes 155,392 test sequences from the Rfam-60 dataset while the negative (i.e. out-of-distribution) set contains all sequences from untrained miRNA families and randomly selected sequences from all other types of ncRNA in Rfam. There are 186,112 sequences in the out-of-distribution set. For each test sequence, the softmax layer will output a vector of normalized probabilities for all the 165 classes. The test sequence is assigned to the class with the the highest probability in the vector. We will set a threshold on this value so that a test sequence with maximum softmax output below this threshold will be rejected. We empirically determined the threshold by analyzing the distribution of the maximum softmax values for each input sequences.

**Fig. 7:**
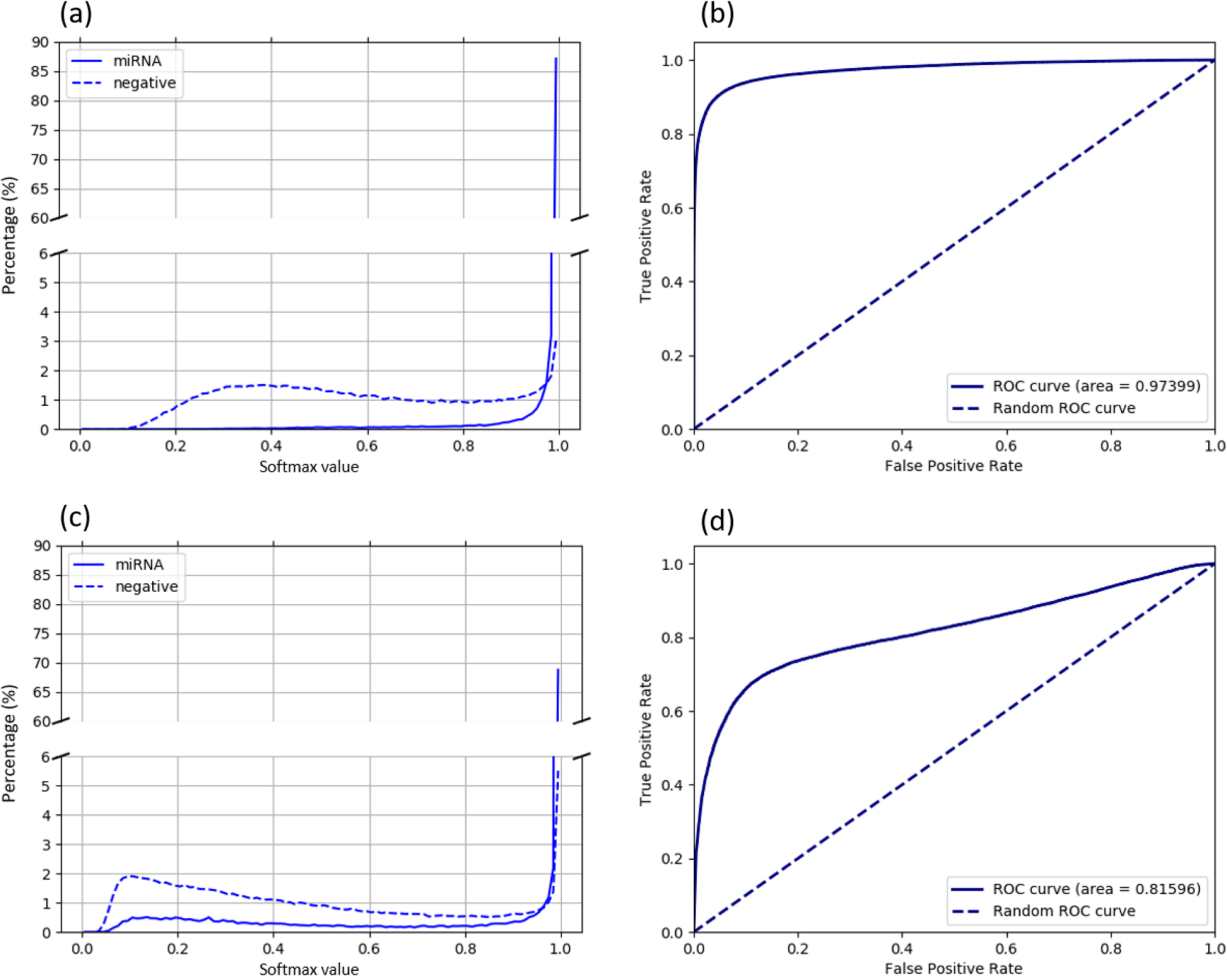
Choosing appropriate softmax probability threshold to reject out-of-distribution samples.

We first plot the distribution of softmax values of the targeted miRNAs and other ncRNAs. Then we show the receiver operating characteristic (ROC) curve, which is constructed using *false* *positive* *rate*$\left (FPR=\frac {FP}{FP+TN}\right)$ and *true* *positive* *rate*$\left (TPR=\frac {TP}{TP+FN}\right)$ computed under different thresholds. Figure 7a and c show the distribution of the softmax probabilities for targeted miRNAs and negative samples. The comparison of (a) and (c) shows that using one-hot encoding matrix leads to smaller overlaps between the two distributions, which is consistent to the comparison of the ROC curves in Fig. 7b and d. Most of softmax values of the targeted miRNAs are greater than 0.9 and the area under the ROC curve for one-hot encoding matrix is very close to 1. By using one-hot encoding matrix, we can find an appropriate probability threshold to reject a majority of the negative samples (high precision) while still keeping targeted pre-miRNAs (high sensitivity). According to Fig. 7b, we choose the threshold leading to a large F-score. The default softmax value threshold for our trained CNNs is 0.977, with associated FPR of 0.05. Any test sequence with maximum softmax probability below 0.977 will be rejected.

**Fig. 8:**
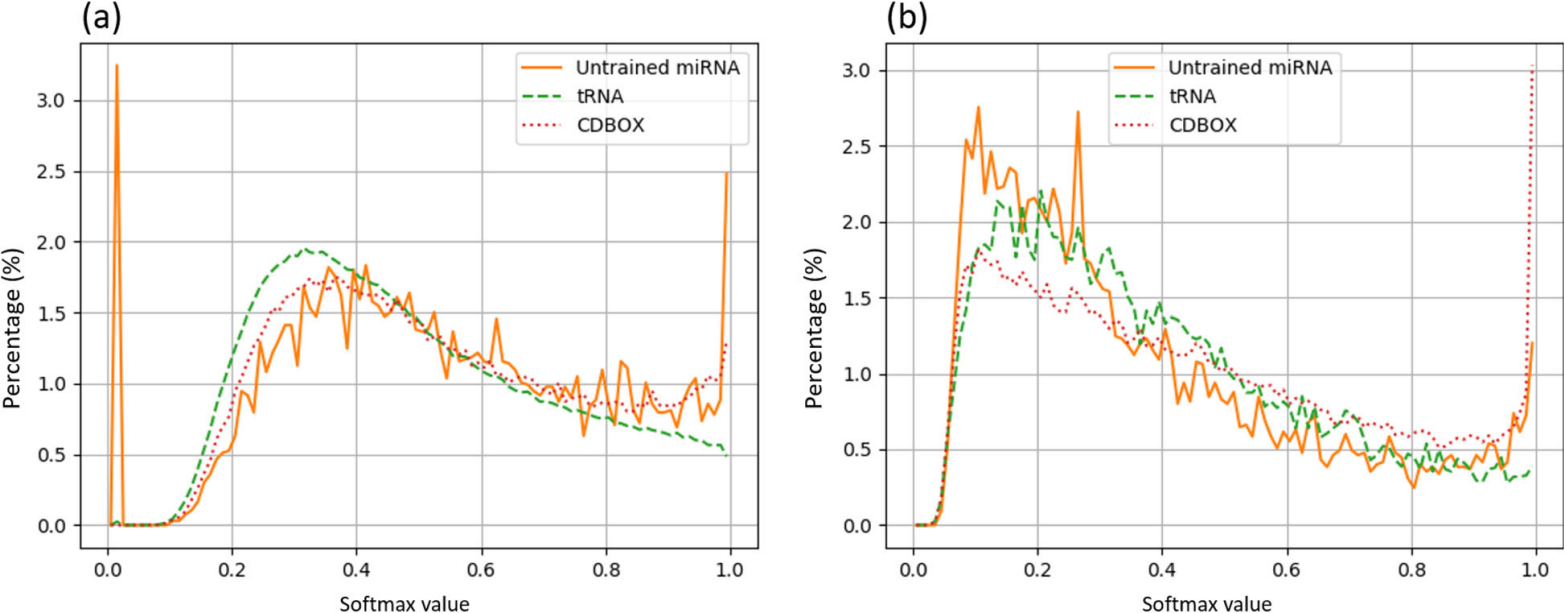
Distribution of softmax values for unseen miRNAs, tRNAs, and C/D box snoRNAs. In both plots, the bin width is 0.01. (**a**) uses the one-hot encoding matrix model; (**b**) uses the pair matrix model

We hypothesized that using pair and probability matrix cannot distinguish different pre-miRNA families because of their similar secondary structures. These matrices should thus be able to distinguish different types of ncRNAs with different secondary structures. Thus, we constructed a smaller negative data set containing tRNA, C/D box snoRNA, and other unseen miRNA families, including 20,000, 60,000 and 6,500 sequences, respectively. The secondary structure of tRNA is cloverleaf, which is very different from miRNA’s hairpin structure. But the C/D box’s stem box structure is somewhat similar to miRNA’s. According to Fig. 8b, probability/pair matrix can distinguish tRNA from miRNA well, but still has difficulty rejecting C/D box snoRNAs. Considering that different types of ncRNAs might share globally or locally similar structures, pair and probability matrices have limited utilities in ncRNA classification.

### Directly classifying mature miRNAs

As many small RNA-seq datasets contain only mature miRNA, we evaluated whether deep learning could be used to directly classify mature miRNAs. As mature miRNAs in the same family can be well conserved because of their binding preference, using either mature miRNAs or pre-miRNAs as the training data may lead to similar classification accuracy for mature miRNAs. We again conduct the comparison using Rfam-60 set, where 50 sequences are used for training and 10 for testing. As we cannot conveniently obtain the mature miRNA annotation in the pre-miRNA families in Rfam, we downloaded the mature miRNAs from MiRBase. Thus, two CNN models are trained on pre-miRNAs and mature miRNAs, respectively. All the test sequences are mature miRNAs. For all the sequences, only one-hot matrix is used because of its superior performance. The mature miRNA classification accuracy of using pre-miRNAs and mature miRNAs as training data is 65.26% and 92.43%, respectively. Thus, when there are no reference genomes and read mapping cannot be used to identify possible pre-miRNAs, mature miRNAs should be used as training data for CNNs.

### Performance on the input sequences with extra bases

Determining the exact boundary of pre-miRNAs in genomes is still challenging. For example, reads from small-RNA seq data can be mapped to reference genomes to identify possible mature miRNAs. Then those regions plus possibly mapped miRNA regions will be extended to identify candidate pre-miRNAs. The extension can go beyond the true pre-miRNA boundaries. Thus, we investigate whether having extra bases affects the classification accuracy. We still use Rfam-60 as our dataset, but 5, 10, 15 or 20 random nucleotides are added around each test sequence. The results can be found in Table 4.

**Table 4:** Classification performance on the test sequences with added bases

Number of added bases	Accuracy	F-score
5	97.52%	97.63%
10	96.88%	97.16%
15	95.47%	95.27%
20	94.70%	94.48%

### Comparison with other tools

In addition to the classification accuracy, the running time is also an important consideration for practical applications, especially when identifying miRNAs from next-generation sequencing data. Here, we compared the classification accuracy and running time of our trained CNNs with Infernal and miRClassify [45]. We also evaluated the performance of each method as the number of miRNA families (i.e. classes) increased. Four testing dataset were constructed by randomly selecting 1000 sequences from Rfam-300, Rfam-120, Rfam-60, and Rfam-30 respectively. Note that all these testing sequences are chosen from the set excluding training sequences and thus have no overlap with the training data for our CNN models. This experiment was repeated for five times and the average performance was reported in Table 5. The variance of each experiment in one-hot matrix method and Infernal is very small (less than 5*e*- 3). And for the miRClassify, the variance is slightly bigger and the biggest variance is 0.02. In order to run Infernal, we directly downloaded the covariance models associated with the corresponding dataset from Rfam. Thus, it is possible that some of these test sequences were used for training the covariance models. MiRClassify uses a hierarchical random forest model to classify the miRNAs into different families. The models of MiRClassify were downloaded from their website and they were constructed from miRBase version 16.0.

**Table 5:** Comparison with Infernal and miRClassify

Tool	Rfam-300		Rfam-120		Rfam-60		Rfam-30	
	Acc.^1^	Time^2^	Acc.	Time	Acc.	Time	Acc.	Time
one-hot matrix	98.94	4.52	98.60	4.53	97.86	4.52	97.45	4.54
Infernal	98.30	265.92	99.06	322.43	99.34	405.15	99.42	486.78
miRClassify	36.50	250.53	46.23	252.76	48.24	254.56	48.80	258.12

^1^Acc. refers to accuracy (%).

^2^Time refers to running time (s)

To ensure a fair comparison in the running time, we used single core for all the three tools because miRClassify is single-threaded. For Infernal, we set the option ‘–cpu’ as 1. All other options for Infernal are the default parameters. The command is:

>*cmscan –cpu 1 rfam_60.cm rfam_60.fa*

Here, ‘rfam_60.cm’ contained all the required covariance models and ‘rfam_60.fa’ is the test sequence set. For each query sequence, Infernal might generate several hits. In that case, we only kept the one with the lowest E-value. CNN model was implemented by Keras so we added extra commands to make sure only one core was used. In addition, the mini-batch size used in CNN was 64. Table 5 summarized the results.

The result in Table 5 shows that despite the possible overlaps between training and testing data for Infernal and MiRClassify, our trained CNN models still have high accuracy with minimum running time. We then conducted the *χ*^2^-test between the 20 accuracy values output by the three methods. The *p*-value between the one-hot matrix method and Infernal was very close to 1 (0.999), indicating that their accuracy is comparable. On the other hand, the *p*-value between ours and miRClassify is 4.59*e*- 275. The running time comparison also shows that Infernal took more time as the number of families increased. The other two methods were not affected by the number of families.

### Frequently activated filters represent part of mature miRNAs

**Fig. 9:**
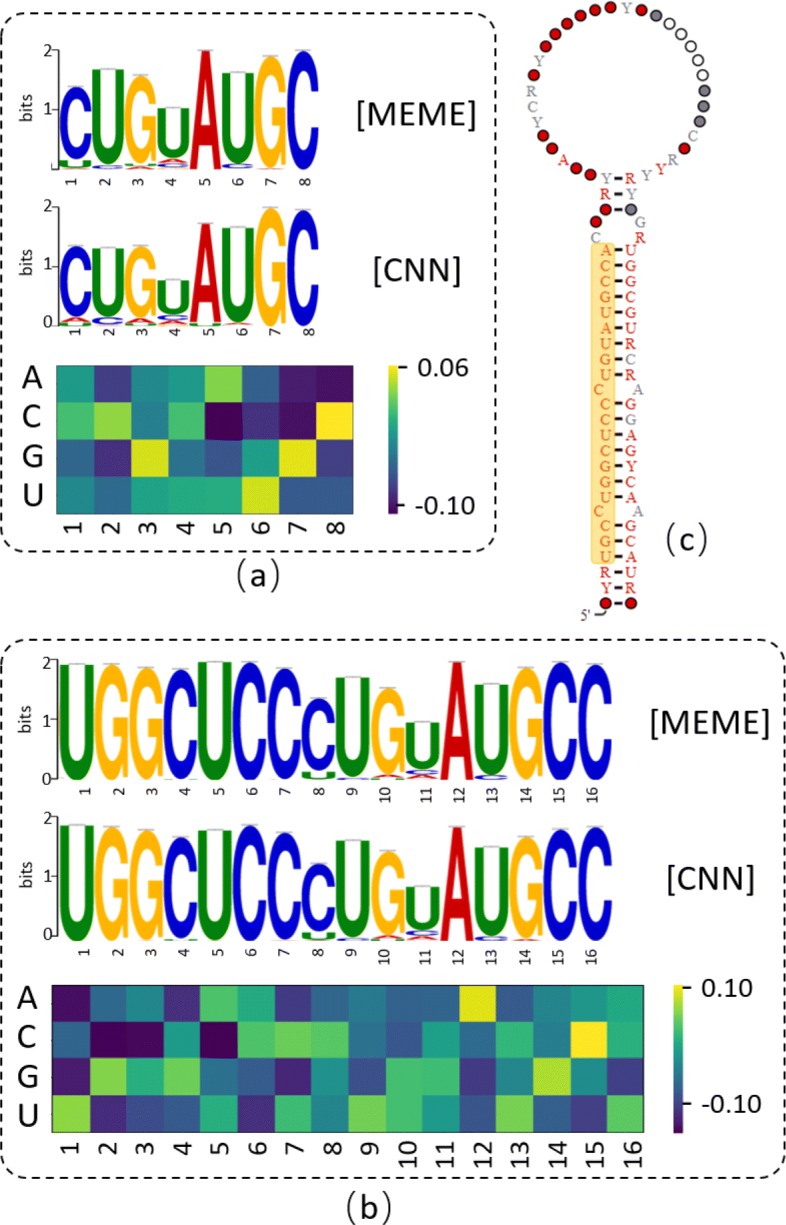
Visualizing and comparing the motifs extracted by MEME [46] and CNN model in RF00247. (**a**) Motifs extracted by MEME and CNN and the corresponding convolution filter of length 8. (**b**) Motifs extracted by MEME and CNN and the corresponding convolution filter of length 16. (**c**) The secondary structure of RF00247 with highlighted mature miRNA

To interpret why the one-hot encoding method performed well, we visualized some motifs extracted by our CNN model. Employing the method used in DeepFam [29], we utilized the most frequently activated filters in trained Rfam-300 model to extract motifs from the RF00247 training sequences. We compared the motifs obtained by CNN with the motifs produced by MEME on training sequences, as shown in Fig. 9. Because the convolution layer used filters of different sizes, this model can identify motifs with various lengths. We found that the identified motifs represented part of the mature miRNA. We tested other families and had the same observation. This is consistent to the findings by DeepFam.

## Discussion

We evaluated and compared the classification performance using different encoding methods and CNN architectures. Based on the experimental results, simple one-hot matrix performed much better than other encoding methods that explicitly incorporate predicted secondary structures. This could be caused by similar secondary structures among different types of pre-miRNA families. As shown by Do et al. [37], it is possible that encoding secondary structures will benefit distinguishing miRNAs from other ncRNAs in the binary classification problem.

In practice, input data such as small RNA-Seq can contain sequences from other types of ncRNAs. Useful miRNA classification must be able to reject out-of-distribution samples. Our experiments demonstrated that using softmax output can achieve an optimal trade-off between sensitivity and precision in distinguishing targeted miRNAs from other sequences. Thus, the designed classification models are practically useful in conducting finer scale miRNA analysis. By comparing our tool with a general ncRNA classification tool Infernal and also another machine learning based miRNA classification tool, we conclude that ours can achieve high sensitivity and accuracy with significantly reduced running time.

## Conclusion

In this work, we developed CNN-based classification models for identifying different types of miRNAs. By using the output of the softmax probability as a threshold, our model can reject other types of ncRNAs and out-of-distribution miRNAs with high precision. Comparing with two existing methods, our one-hot encoding method takes much less time and still has high accuracy.

Although this work only concerns miRNAs, the trained CNNs can be extended to classify other types of ncRNAs. The method holds the promise to achieve comparable performance while achieving significant speedups compared to Infernal. It is our future work to extend and optimize our model for other types of ncRNAs.

## Data Availability

The source code and datasets used during the current study are available at https://github.com/HubertTang/DeepMir

## Abbreviations

CNN: Convolution neural network
FPR: False position rate
LSTM: Long short term memory
miRNA: microRNA
MFE: Minimum free energy
ncRNA: non-coding RNA
pre-miRNA: precursor microRNA
pri-miRNA: primary microRNA
rRNA: ribosome RNA
ROC: Receiver Operating characteristic
tRNA: transfer RNA
TPR: True positive rate
